# Smart Environments and Social Robots for Age-Friendly Integrated Care Services

**DOI:** 10.3390/ijerph17113801

**Published:** 2020-05-27

**Authors:** Ionut Anghel, Tudor Cioara, Dorin Moldovan, Marcel Antal, Claudia Daniela Pop, Ioan Salomie, Cristina Bianca Pop, Viorica Rozina Chifu

**Affiliations:** Computer Science Department, Technical University of Cluj-Napoca, Memorandumului 28, 400114 Cluj-Napoca, Romania; ionut.anghel@cs.utcluj.ro (I.A.); dorin.moldovan@cs.utcluj.ro (D.M.); marcel.antal@cs.utcluj.ro (M.A.); claudia.pop@cs.utcluj.ro (C.D.P.); ioan.salomie@cs.utcluj.ro (I.S.); cristina.pop@cs.utcluj.ro (C.B.P.); viorica.chifu@cs.utcluj.ro (V.R.C.)

**Keywords:** social robots, ambient assisted living, machine learning, older adults care, daily life activities monitoring, technology limitation and acceptance, care services models

## Abstract

The world is facing major societal challenges because of an aging population that is putting increasing pressure on the sustainability of care. While demand for care and social services is steadily increasing, the supply is constrained by the decreasing workforce. The development of smart, physical, social and age-friendly environments is identified by World Health Organization (WHO) as a key intervention point for enabling older adults, enabling them to remain as much possible in their residences, delay institutionalization, and ultimately, improve quality of life. In this study, we survey smart environments, machine learning and robot assistive technologies that can offer support for the independent living of older adults and provide age-friendly care services. We describe two examples of integrated care services that are using assistive technologies in innovative ways to assess and deliver of timely interventions for polypharmacy management and for social and cognitive activity support in older adults. We describe the architectural views of these services, focusing on details about technology usage, end-user interaction flows and data models that are developed or enhanced to achieve the envisioned objective of healthier, safer, more independent and socially connected older people.

## 1. Introduction

According to the World Health Organization (WHO) the proportion of people over 60 years will double from 11% in 2006 to 22% by 2050 [1]. In this context, the world is facing major societal challenges because the aging population is putting increasing pressure on the sustainability of care. First, the demand for care and social services is steadily increasing while the supply is constrained by the decreasing workforce capacity. Second, as people grow older, the costs of care rapidly increase—mainly due to chronic diseases and comorbidities management.

To address this, WHO launched a worldwide initiative “age-friendly cities and communities” with a view of transforming and tailoring living spaces and cities to the needs of older adults [2]. The proposed age-friendly cities model guides the implementation of new services and policies in various domains from communication and information up to housing or community support and health services. The development of such a model was driven by several factors, such as improving the environments in which the older adults live and developing community-oriented policies promoting opportunities for enhancing the quality of life of older adults [3]. At the same time, recent technological advancements such as ambient intelligence and information and communication technology (ICT) mediated intervention, have supported the model implementation offering opportunities for older adults to be proactive in addressing age related challenges and co-producers of new services development. In fact, the model checklist of essential features of age-friendly cities provides lots of references to the adoption of new technological solutions [4].

Recent studies seek to extend the age-friendly cites model with the technology enablers needed to make it compatible with the smart and age-friendly ecosystems [5]. The direction of developing smart houses or indoor environments for older adult care [6] is identified as a relevant one as it optimally integrates the technology with the domain of housing defined by in the WHO age-friendly cities model [7]. In this context the implementation of smart, physical, social and age-friendly environments is a key intervention point for enabling older adults to stay as much possible in their residences and delay their institutionalization. Nevertheless, in this case, there is a high risk that older adults may become socially isolated and will not be able to care for themselves. If certain daily life activities (i.e., eat, drink, take medication as planned, move, etc.) are not properly carried out will significantly impact their condition, speeding up the deterioration of their health and wellbeing—especially in case of older adults suffering from comorbidities. However, the recent adoption of virtual personal assistants and/or socially assistive robots is advocated as a potential technological solution that may positively impact the older adults in society. Many of their features empower the older adults to remain socially engaged and at the same time they may successfully mediate the delivery of timely healthcare interventions [8]. Social robots may not only support older adults with daily activity, but at the same time they may contribute to emotional wellness by keeping older adults socially engaged and activating social interactions in communities [9]. Moreover, they may not only drive the implementation of new services in various domains defined by WHO’s age-friendly model in which older adults perceive the social robots as helpful companions, but they also promote the human-to-human interactions.

Thus, although independent living reduces the demand and costs for care, it can also reduce the quality of care if not supported by innovative ambient assistive living (AAL) technologies and socially assistive robots. Efforts need to be committed to integrate and further develop such technologies to realize advanced care services for prolonging the autonomy and well-being of older adults. The implementation of technology-enabled supportive intelligent environments may offer opportunities to promote improved and more personalized care services into older age. Combined with care functionalities such as remote care support, medical reminders, behavioral monitoring and virtual coaching, they have the potential of delivering the right support to the end-users when help is needed. The combination of activity, safety, social and comfort functionality can lead to an extension of the time spent living in their own home by shifting or postponing parts of in-patient (i.e., care facility services) treatment, therapy and exercise training into the own home; thereby it will directly decrease or delay admissions into a care institution, diminishing the use of professionalized care services and/or lowering the burden of health care services and facilities.

In this context, the smart and ‘age-friendly’ environments will become fundamental pervasive technologies for supporting older adults care while the social robots may become important intervention tools due to their ability to provide support to older persons and open links with their community and professional caregivers and to cooperate and interact with older adults engaging them in the care process. Starting from the above identified challenges, the contribution of this study is twofold:

- A survey of smart environments and robot assistive technologies that have the potential of supporting the independent living of older adults at home by implementing age-friendly care services. In this process we identify the challenges in implementing the new care service models, existing technology limitations and its acceptance by the older adults;
- A discussion on how these technologies are used for the development of two care services for older adults centered and integrated care polypharmacy management and control of wellbeing decline by social and cognitive activity engagement.

The study is structured as follows: Section 2 reviews existing smart environments and social robots’ technologies for older adult care, Section 3 analyses the application of the technology for new integrated care services and finally Section 4 presents the conclusions of the review.

## 2. Smart Environments and Assistive Robots’ Technologies Review

In this section we provide an overview of state-of-the-art assistive technologies focusing on three main directions: objective monitoring using sensors devices, data analysis and machine learning for behavior assessment and finally delivering interventions using social robots. In the end, we discuss for each type of technology the limits, challenges and adoption from the perspective of older adults.

### 2.1. Monitoring Daily Life Activities

The development and deployment of sensors and smart devices for monitoring older adults’ activities is a dynamic research and innovation area currently triggered by the advent of big data and machine learning technologies. On top of the monitoring devices, applications are developed for assisting the older adults’ healthcare, improving the social communication and inclusion or for managing various activities targeting to prolong independent living. In [10] the most important dimensions of older adults’ lifestyle that need to be properly monitored are identified as: the physiological parameters and the body functions, the changes in the daily life activities (DLAs) and daily routines, the social factors and the environmental factors. In [11] and [12] the data to be monitored and collected for older adults’ support and intervention is classified in behavior, health, occupancy, lifestyle, home automation, environmental and personal safety. Similarly, in [13] the most common data types to be monitored and used in AAL systems are identified as: behavioral habit data, physiological information, healthcare information and environmental data.

On other dimension, there are several types of sensors which can be used to monitor and analyze different aspects of the older adults’ healthcare and well-being, most frequently being classified in: (a) physical sensors, (b) virtual sensors and (c) logical sensors.

Physical sensors are the most frequently used type of sensors in collecting data about the older adults’ healthcare and well-being. Table 1 presents an overview of various AAL physical sensors and the type of monitored data that was identified in the research literature [14]. These research approaches are classified and discussed below.

Wearable physical sensors are used to collect the data about (i) the physiological signs of a person (e.g., heart rate, blood pressure or temperature, blood cholesterol, blood oxygen saturation, respiratory rate, blood sugar level, etc.), (ii) the posture, gait and movement, (iii) the social interaction or (iv) the activities performed by a person during a day. All these data are analyzed to assess the health/wellbeing status of a person [15,16,17,18,19], the sleep quality [20,21,22,23] and the level of stress and cognitive decline [24,25,26] or to detect the falls [27]. Currently, the most important wearable sensors that can collect this type of data are embedded into wristbands, smart clothes, smartphones or smartwatches, arm bands or chest straps.

Monitoring the physiological signs and physical position provides valuable information regarding the health status of a person. Some of the main benefits provided by the wearable sensors in monitoring the health status of a person are the following [28]: (i) allow to continuously monitor the vulnerable patients, (ii) allow the medical specialist to have a better view on the patient’s health issues and to make the most accurate diagnosis and (iii) offers the patients the opportunity to evaluate their health condition and consult a doctor when appropriate. In [15] the data collected to evaluate the health status of a patient is: physiological signs and physical position. The types of sensors used for collecting the data are airflow sensor, galvanic sensor, body temperature sensor, blood oxygen sensor, electroencephalogram (EEG)/ heart rate sensor and digital accelerometer. An Internet of Things (IoT)-based system for monitoring the health status of Cardiovascular disease(CVD) patients consisting of a sensing layer responsible for collecting the physiological signs of the patient (e.g., blood pressure, electrocardiogram (ECG), amount of oxygen in the blood (SpO2)) and the patient’s location is proposed in [16]. Custom wristbands that integrate specific sensors (i.e., piezoelectret sensor) that can detect heartbeats are also proposed [29]. The piezoelectret sensor can help detecting the pulse waveform, which is similar to the one provided by an electrocardiogram. Similarly, arm-wearable ECG sensors are researched for monitoring the heart rate that integrates the main components of an ECG [17]. The sensor provides two functions, namely a monitoring function in which the signal is acquired and a Holter function in which the signal is stored in the internal memory. Authors of [18] develop a smart t-shirt for recording the ECG signals which integrates a set of active electrodes and an ECG Portable recorder. The accuracy of ECG signals recorded with the developed device was comparatively evaluated with a standard 12-lead Holter. A system for monitoring in real time the CVD patients’ heart rate, blood pressure and body temperature which of multiple wearable sensors and collects medical information about patients with CVD, is connected to a web portal for displaying the acquired data from patients in [19].

Sleep quality is an important factor for a person’s stage of health because it reduces the risk of developing chronic diseases and mental disorders. Most state-of-the-art approaches for monitoring sleep quality focus on comparative analyses and evaluation of the commercial devices available on the market [21,22,30], while only some of them present academic research solutions [20,23,31]. For example, in [21] commercial devices for monitoring the sleep quality are analyzed, the Up Move Jawbone (U) and the Withings Pulse accelerometers (monitor sleep duration, how many times s/he wakes up at night, etc.), the Bodymedia SenseWear Pro Armband actigraph (monitors the sleep quality by estimating the sleep duration and efficiency) and the home-polysomnography (collects data about thoracic and abdominal movements, airflow, etc.). Similarly, authors of [22] evaluate five wearable devices for recording the sleep quality, namely the Basis Health Tracker, the Misfit Shine, the Fitbit Flex, the Withings Pulse O2 and the Actiwatch Spectrum actigraph. The Basis Health Tracker is an actigraph embedded in a wristwatch, while the Misfit Shine is a sleep-tracking device provided with a strap to be worn on the hand. The Withings Pulse O2 is also a sleep-tracking device worn on the wrist, while the Actiwatch Spectrum is a wristwatch with an embedded accelerometer. In [30] the accuracy of a commercial device, namely, Fitbit Charge 2, compared to polysomnography, in measuring the sleep and the wake states is analyzed. This device can detect, besides the sleep and wake states, the time spent awake, in light or in deep sleep as well as to track the level of daily activities. In [23] it is proposed a wearable device for monitoring the abdominal and thoracic respiration that can be used to detect the obstructive sleep apnea by monitoring the breathing status. Chest-worn sensors collect data about the posture (during the day or the sleep), the position changes and body acceleration of a person in order to detect the sleeping and the waking periods to detect the quality of the sleep during the night and the time spend by the person in bed [20]. In [31] wearable sensors positioned on arms and chest are used to monitor the sleep quality. The sleep quality is evaluated based on the sleep posture and the sleep stages (awake, rapid eye movement (REM) sleep and non-REM sleep cycles).

Monitoring the level of stress is very important because, on long term, it has a negative impact on the cognitive functions of a person. Also, by monitoring the cognitive function, valuable information is obtained that can be used to improve the daily/detect early cognitive decline in the case of older adults. In [24] the physical attributes (e.g., galvanic skin response and skin temperature, EEG heart rate, respiration rate and voice data) are monitored and collected in order to assess in real time the emotional, physical and mental stress of a person. Data are acquired with a set of wearable biosensors integrated in smart clothing and with a microphone. In [25] a smartwatch which integrates a Global Positioning System (GPS) sensor, an ambient light sensor and an acceleration sensor, is proposed as a solution for monitoring wandering risk for older adults suffering from dementia. The developed smartwatch acquires the following types of data that are used to avoid the risk of the patient losing their way when they experience wandering episodes: the older adult position and the route in which s/he moves that is necessary to identify if the older adult moves outside the area considered to be safe, the sunlight exposure duration and the number of older adult’s steps. In [26] the benefits of the physical activity on the cognitive functions of the older adults are inferred based on (i) the data (e.g., light/ moderate to vigorous physical activity) collected with an accelerometer and (ii) a set of questionnaires containing information about the cognitive ability of older adults. A platform for monitoring older adults at home in order to detect the early cognitive decline is researched in [32]. It integrates Infrared (IR) motion sensors with magnetic contact sensors and sensors for monitoring user computer interactions (e.g., mouse movements, computer usage time, etc.) and uses all the collected data to build the profile of changes in the activities performed by older adults, which are than analyzed in order to detect possible cognitive decline.

In the case of fall detection, most approaches from the research literature are based on accelerometers and gyroscopes. Accelerometers can provide information about the motion data during daily living activities (i.e., walking, running, stepping and falling), as well as the instability that can appear during these activities and can provide valuable information in predicting the risk of falling during walking [27,33]. Usually a wearable sensor feds data to a fall detection algorithm to identify the fall risk within the daily activities of a person [34]. In [35] the data about acceleration signals, postural instability and falls are recorded with a tri-axial seismic acceleration sensor placed on a belt while [36] presents an approach for detecting the fall, based on a smartphone which integrates a tri-axial accelerometer and a tri-axis gyroscope and is used to record the acceleration patterns, in the case of older adults, for the following types of daily activities: sitting, lying, jumping, running, walking and hitting the sensor. A fall monitoring system which integrates a portable sensor that can be placed on the person’s shoulder, waist or foot and a mobile phone is proposed in [37]. The portable sensor records the data with a triaxis accelerometer, a triaxis gyroscope and a triaxis magnetometer and sends it to the mobile phone. The collected data are used to compute the acceleration and Euler angle which are provided as input to a fall detection algorithm.

In contrast with wearable sensors that need to be placed on the body of the person, ambient (non-wearable) sensors are positioned in different places of the older adult’s home and are used to collect data regarding the behavior, the occupancy, the lifestyle, the environmental safety and the personal safety of a person. The non-wearable sensors can be used to detect the well-being/health status of a person as well as to monitor the sleep quality.

Ambient sensors can be used for monitoring the daily living activities performed by older adults as well as their daily routine and to detect behavior changes caused by the deterioration of the health state. In [14] the data that are monitored and collected in order to evaluate the wellness of the older adult in a controlled environment (i.e., his/her house) are: behavior, occupancy, lifestyle, environmental safety and personal safety. The monitored infrastructure integrates the following types of non-wearable heterogeneous sensing units installed in the older adult’s houses: (i) movement sensors, (ii) electronics and electrical devices monitoring sensors placed on the electrical and electronic appliances, (iii) toilet sensors, (iv) contact sensors to monitor the opening/ closing of a door, office desk or and self-grooming table and (v) force sensors placed on sofa /bed/ armchair to monitor the sleeping or sitting activities. In [38] the solution proposed for detecting the deviation from the daily activity routine of older adults is based on a non-intrusive monitoring infrastructure consisting of low-cost sensors such as passive infrared (PIR) motion detectors and magnetic door contacts sensors located in each rooms of the house. The monitored data contains information about the older adults’ lifestyle and is used to detect unusual activities performed by an older adult in a day or deviations from his previous routine.

Smart beds as well as cameras are an alternative to the wearable commercial devices for monitoring the sleep quality, beside the polysomnography and the videosomnography that can be used in clinical evaluations. Authors of [39] propose a non-intrusive monitoring infrastructure consisting of pressure sensors embedded in bed which is used for detecting sleep quality. The sleep quality is assessed based on the sleep position and the sleep stages. In [40] a microbend fiber optic mat embedded in the mattress of the bed is used to monitor the sleep quality. The sleep quality is evaluated based on the following types of data acquired from sensor: sleep duration, movements during the sleep, heart rate, awake stage duration, time spend in bed as well as the respiration rate. Similarly, in [41] a sensor integrating an Emfit foil electrode embedded in the mattress of the bed is used to measure the sleep quality. The sleep quality is evaluated based on the movement activity and the heart-rate fluctuations. Piezoelectric film sensors can be embedded in the bed mattress to monitor the sleep quality, based on the variation of the heart and respiration rate and the binary actigram [42]. Microsoft Kinect camera can be employed to detect the human shape and the body movements and a sensor tag that provides information about the sleep environment such as the temperature and humidity [43], while Near-Infrared camera can be used to analyze the sleep behavior, based on the collected videos/images [44].

Non-wearable sensors for fall detection approaches that are based on ambient sensors use cameras, infrared sensors, acoustic sensors or force sensors installed in the home environment to detect the fall. In [45], an approach for detecting the fall that is based on low-cost fall detector that integrates Raspberry Pi 2 and an universal serial bus (USB) camera and several algorithms (e.g., background subtraction, Kalman filtering optical flow) is proposed. The detector is developed as a portable device that can be moved from a room to another of the older adult’s house and can detect the following positions: walking, standing, sitting and falling. Similarly, in [46] is presented a method of detecting the fall in the case of older adults. The method is based on a Microsoft Kinect sensor for collecting the images combined with an algorithm for background subtraction and an ensemble of decision trees. In [47] is proposed an approach for detecting the fall in the case of older adults that is based on a ground sensors network consisting of accelerometers and force sensors installed on the tiles from each room of the older adults’ apartment while [48] proposes a classification method which is able to detect between human fall from non-fall, based on the acoustic waves transmitted on the ground collected with a floor acoustic sensor. A method to detect the fall in the case of older adults which combines acoustic sensors that collect the sound signals of the footsteps performed by older adults during the daily activities and a support vector machine algorithm is used to make distinction between fall and non-fall sounds is proposed in [49]. Authors of [50] develop a fall detection system that uses a circular array of 8—microphones to detect the older adult’s fall in real time and sends alerts to the caregivers.

Wearable sensors can be combined with ambient sensors in complex monitoring infrastructures that can be used to obtain more accurate results in monitoring the heath/well-being status or sleep quality. In [51] the data that are monitored and collected in order to evaluate the wellness of the older adult in terms of functional and cognitive capacity can be classified in: vital sign, social interaction and physical activity. The monitoring infrastructure consists of a wireless sensor network integrating a collection of wearable and non-wearable heterogeneous sensors. eWall [52] is a holistic monitoring platform for home, which collects the following type of data in the case of older adults suffering of chronic obstructive Pulmonary disease, mild cognitive impairments or others age-related impairments: (i) health data (e.g., SpO2, pulse, heart rate), (ii) well-being state data (physical activity, sleep, mood), (iii) environmental data (e.g., temperature, luminosity). In [53] the sleep quality is monitored with a wearable three-axis accelerometer and a pressure sensor installed in bed. The accelerometer is used to determine the sleep pose and the sleep state (e.g., REM sleep and non-REM sleep cycles), while the pressure sensors are used to detect heart/respiration rate. Based on the collected data a novel algorithm is develop that is able to detect the sleep quality, based on the number of apneic episodes, the duration of sleep and the depth of sleep.

Virtual sensors are a source of data coming from software applications or services. For example, it is possible to determine an older adult’s location not only by using tracking systems (physical sensors) but also by using a by browsing an electronic calendar, a travel-booking system, emails etc., for location information. Other attributes that can be sensed by virtual sensors include, e.g., the user’s activity by checking for mouse-movement and keyboard input. Logical sensors are an extension of the virtual ones and make use of a couple of information sources and combine physical and virtual sensors with additional information from databases or various other sources in order to solve higher tasks. Pain Care is a healthcare app developed for iOS and Android device that allows older adults to keep a medical journal in which stores information about the medication that s/he take, specific symptoms/pains or side effects of medicine that could occur during the treatment [54]. Based on this medical journal, the causes of pain and treatment efficacy is estimated. All these data are transmitted to medical specialist that can adjusted the medical treatment according to the patient personal profile and his medical journal. Researchers have investigated the benefits of using computer or mobile applications (e.g., memory training application or brain training applications) in maintaining/ improving the cognitive functions of older adults [55,56,57,58,59,60]. In [55,56] specific smartphone applications for improving the cognitive function of older adults are comparatively analyzed. The attention and the working memory are improved by engaging the older adult in training tasks with different difficulty levels for concentration, speed, memory, visual and logic. HealtheBrain, is another smartphone application in which physical activities are combined with memorizing activities in order to improve the cognitive functions of older adults with and without mild cognitive impairment [57]. Similarly, in [58] the results of using the cognitive training game in improving the cognitive functions of older adults is analyzed. The game includes a set of tasks to be performed by the older adults which aim to train various cognitive functions of the older adult, such as attention, memory, visuospatial or language function. There are also studies that demonstrate the efficiency of using computer/smartphone applications in dementia treatment. For example, in [59] three smartphone applications are analyzed: EVO which is a cognitive training application that reduces the symptoms of depression, iPST which acts as a psychotherapy for depression and health tips which assists in the treatment control. In addition, in [60] the effect of playing action video games in increasing the cognitive ability in the case of the people suffering of with dementia is analyzed. Reference [61] presents SONOPA, a framework which combines ambient sensors with social networks to create social connection between older adults, based on their hobbies, localization or activity levels. The activity levels and the number of persons in a house (i.e., the level of occupancy) are determined based on the data collected with PIR and visual sensors, while the socialization interaction level of an older adult in a day is computed based on the level of the occupancy of the house and the information collected from the social network (the message communication, the visualized photograph, etc.). In addition, a matching algorithm is proposed that can identify new social connections for the older adults with low social level based on the data collected from sensors and from the social network (i.e., person profile, socialization level, etc.).

Considering the above, we have mapped the nowadays available sensing devices onto different type of assessments they enact aiming to determine their potential usage as reliable source of data for deciding on robot-based interventions (see Table 2).

### 2.2. ML for Behavior Assessment

Machine learning (ML) techniques are an important component for building smart environments and associated technologies. Using the gathered monitored data, these techniques can be used for identifying and assessing certain situations in the older adults’ behavior, situations that usually require personalized intervention from the caregivers or health professionals [62]. At the same time, the automatic recognition and classification of various daily life activities has the potential of reducing the costs associated with the healthcare of the older adults significantly, especially in the case of those cognitively challenged. By using sensors and advanced analytics over collected data, it is possible to extract information in real time about the monitored person and thus it is possible to detect anomalies and patterns that may indicate wellbeing, social and cognitive decline as well as healthcare problems that require intervention.

In this section we analyzed a selection of representative research articles which consider the application of ML techniques for various cases of behavior assessment for older adults. For each representative research article, the following characteristics are considered: the machine learning techniques used, their advantages and disadvantages and type of behavior assessment targeted. We have identified and classified the most important ML techniques that are used for building such smart AAL technologies:

- Classification techniques—The state-of-the-art literature features several methods based on different types of classifiers for monitored data streams out of which ensemble learning methods are considered the best techniques for the classification of the data streams. There are still a lot of challenges posed by the data streams in the case of the ensemble learning algorithms such as the temporal dependencies [63], the concept drifts [64] and the feature drifts [65] and those challenges may appear especially in the monitoring of the daily living activities that are situation-aware where similar monitored data can correspond to related activities such as ascending stairs or descending stairs;
- Regression techniques—The application of regression techniques for daily living activities recognition in context-aware AAL systems [66] is challenging because the identification of the activities should be performed after the beginning of the activities as soon as possible. A part of the limitations of the current approaches are the recognition of the activities after they are completed and the training of the models using offline historical data, a machine learning phase that leads to models which cannot predict the ongoing activities in a timely manner;
- Clustering techniques—The clustering of the data streams should be adaptable due to the fact that the underlying data streams may change and evolve significantly in time, like in the case of data that results from the monitoring of the older adults while they perform different types of daily living activities. In [67] are addressed in more details challenges regarding the clustering, the labeling and the interpretation of the IoT data streams dynamically, challenges that exist especially in those AAL systems that monitor the daily behavior of the older adults;
- Other ML techniques—This category considers techniques such as discovery of association rules, patterns detection, anomalies detection, etc. The abnormal human activities are very diverse [68] in nature due to a variety of aspects such as the way in which the anomalies are defined, the feature representations of the anomalies and the characteristics of the daily living activities data. The detection of the anomalies using various ML algorithms was approached in the research literature in a few studies such as the one presented in [69] where the analysis of the anomalies is not considered as the main subject of the study, but in relation with the recognition of the daily living activities, the discovery of the behavioral patterns and the decision support.

There are three types of older adult behaviors considered that is usually addressed in the research literature through ML techniques, namely daily activities behavior, agitated and aggressive behavior and medication adherence behavior.

Assessing the daily activity behavior of the older adults is relevant for the detection of the abnormal situations [70]. In [71] the authors propose a generic architecture for the monitoring of the activities in smart homes and approach a large variety of ML techniques such as the classification of the activities, the prediction and the reminding of various activities and the detection of the anomalous patterns. However, that review is focused on the basic daily living activities behavior of the older adults and it considers only partially or not at all other types of behavior such as the medication adherence behavior or dementia specific behavior. ML challenges related to the classification of frequent daily living activities (i.e., eating and drinking) are described in [72]. The authors consider data from three datasets collected using various types of monitoring sensors such as power meter sensors and motion, contact and audio sensors. The tested classifiers were support vector machines (SVM), random forest (RF), Fisher kernel learning (FKL) and hidden Markov model (HMM). A different classification approach in [73] considers classification techniques for the remote acoustic monitoring of the older adults in AAL scenarios of residential scale. The data were collected from wireless acoustic sensors and the decision to trigger or not an alarm is taken after the running of a two-stage ANN-based classification process for audio events. One ML usage in AAL systems is the prediction of the daily living activities of the older adults [74] ranging from regression techniques related to the prediction of the CO_2_ consumed inside a room using an Artificial Neural Network (ANN) Levenberg–Marquardt (LMA) prediction model to other ML techniques related to the detection of the daily living activities from data collected by humidity, temperature or microphones [75]. The authors of [14] consider a smart aging system for distinguishing the variations from the baseline. The proposed ML method consists of preprocessing, segmentation, feature extraction, classification, pattern recognition and anomaly forecasting. Moreover, this approach also considers the medication activity with respect to the food intake activity. However, the performance results are affected by noise that can be generated by faulty sensors or by the presence of a visitor. Another approach, [76], proposes a method based on a deep convolutional neural network (DCNN) in order to classify ten types of activities. Even though that approach is based on one of the most preferred solutions for daily living activities monitoring, namely the unobtrusive activity recognition and returns a very high F1 score for eight out of ten activities that are monitored it may be more expensive in terms of computational resources than other approaches. The recognition of the activities was approached in [77] using data collected from public available datasets which are characteristic to smart home scenarios. In addition to classical daily living activities such as bathing, sleeping and eating, that approach also considers the medication intake activity. The proposed classifier is a long short-term memory (LSTM) model and compared to other existing machine learning models it returns better results while the performance of the approach is affected by various dimensions such as the number of residents, the number and the types of activities and the test days duration. The authors of [78] propose a fog-based deep learning fall detection system using data collected by a tri-axial accelerometer. The proposed architecture is based on three layers, namely a medical devices layer, a fog layer and a cloud layer. The results presented in that article show that the deep learning methods such as LSTM and gate recurrent unit (GRU) are better than the classical machine learning methods such as SVM and k-nearest neighbors (K-NN) for falls detection.

Agitated and aggressive behavior is one of the most challenging symptoms of dementia [79] and its automated detection using sensors is useful for the caregivers that can act quickly in these kinds of situations. In [80] the authors consider challenges related to the agitated behavior in the case of the people with dementia. The automatic detection of the agitation is approached considering data from various sensors placed around the bodies of the monitored subjects that collect information about the skin temperature, the skin response and the heart rate and that data are further analyzed using a SVM classifier. One drawback is that the approach requires the sensors to be placed on the bodies of the monitored older adults. The authors of [81] consider the application of novel technologies for the early Alzheimer disease (AD) detection. Some associated symptoms are the aggression, the anxiety, the aberrant motor behavior and the irritability. The data from the subjects was analyzed using an ANN with two classes (control subjects and AD), the approach is low cost and it does not have any side effects. Related to prediction of the agitative behavior for the patients with dementia an important challenge is overcoming weakly labeled and sparse data [82]. The goal is to infer proficiently the agitation episodes from data collected by wearable sensors using multiple-instance learning (MIL) models. However, that data comes only from 10 residential deployments, each with a duration of 30 days. In [83] the data collected from various sensors is used in order to detect the challenging behaviors in the advanced stages of dementia. K-means clustering was applied in order to cluster the residents in two groups, one group characterized by challenging behavior with more passive features, and the other group characterized by challenging behavior with more active features. However, the possibility to detect the disorientation using accelerometer data in different solutions based on assistive technologies is considered as a future research direction. The detection of the agitation behavior of the people with dementia using wearable devices is also approached in [84]. The combination of data from multiple sensors leads to better results than in the case when data from a single sensor is used. The applied classifiers are RF and SVM and the results show that the multi-model sensors are feasible for detecting agitation in the case of the people with dementia.

Medication adherence behavior of the older adults to specific medication plans is critical, especially when the older adults have special conditions such as dementia. The application of pervasive technologies [85] for the monitoring of the daily medication behavior may predict when the medication prompting is effective. The approach presented in [86] considers clustering challenges related to the medication adherence. Since the medication adherence behavior is correlated with other daily living activities such as drinking and eating, that approach also considers other activities. The proposed clustering approach is a k-means that is improved using fuzzy set. The authors of [87] consider the application of reminder-based interventions for the individuals with dementia as the memory limitations often lead to activities that are incomplete or not initiated. The article considers the application of the sensor technologies in combination with machine learning technologies in order to address those challenges. The classifiers considered in that approach are various such as decision trees (DT), K-NN, naïve Bayes (NB), SVM and logistic regression (LR). The approach presented in [88] assesses the medication adherence of 38 older adults with a mean age of approximately 87 years. The two measures of adherence proposed in that article are the percent of the days when the medications were missed and the spread in time when the medications were taken. The data were analyzed using three linear regressions and the medication intake habit was monitored continuously using a MedTracker 7-day pillbox. The medication adherence is approached in [89] in relation with other activities such as walking, drinking water, writing and texting. The applied classification algorithm is RF from Apache Spark. Even though the data were misclassified sometimes using that approach, the application of the near-field communication (NFC) sensors may improve the results. Medication intake behavior in relation with the eating and the drinking behavior is researched in [90]. The experiments were conducted using data collected from gyroscope and accelerometer sensors and the performance of various classifiers such as K-NN, NB, DT, multilayer perceptron (MPC), RF and HMM was evaluated. Moreover, two out of the five analyzed different users were seniors. The results presented in that article are very promising, and the system should provide an adequate basis for smart reminder triggering in the case of the autonomously living seniors. However, that approach was tested only on five seniors the method does not consider special characteristics of the older adults that may have dementia such as memory related problems or unpredictable behavior. The medication intake activities were considered in [91] using a solution based on data collected from a smartwatch in relation with other activities such as texting, writing, walking and bottled water intake. In terms of F1Score, the Gradient Boosted Trees (GBT)-based approach returned better results than the approaches based on LR, SVM and RF. One advantage compared to other approaches from literature that consider the medication intake activity is the fact that it considers at least two types of medication intake. In [92] the medication adherence is considered using a ML approach that is based on body worn sensors. The medication intake activity is analyzed considering correlated activities such as drinking, taking chocolate and eating and the applied machine learning classifier is the DT classifier.

### 2.3. Social Robots Driven Intervention

Social robots are nowadays seen as key technology for supporting older adults care at home or in care institutions. Even from the breakthrough of the assistive robot’s technology two main advantages were identified: the functional capabilities and the affective aspects [93]. They can offer different care functions (physical activity, affective therapy, cognitive training, physiological therapy, etc.) while they can help to increase the quality of life of older adults through companionship and social interaction. There are basically two classes of social robots: physically assistive robots that are focused to perform physical tasks and socially assistive robots that can be used for the social and psychological needs of older adults [94].

Few literature studies offer a clear view over the current status and trends for social robots being focused most on old robot models and their potential application in the older adult care domain [93,94,95]. These studies highlight PARO as one of the first social robots with immediate applicability for older adult care. PARO is an interactive robot developed by Japanese AIST research institute that uses animal therapy as a care method. It has reached its 8th generation with a price around of €5000 and is successfully used in hospitals and care homes across the world, but it can be easily used in the older adult’s home. PARO uses tactile, light, audition, temperature and posture sensors to learn the surrounding and interact with older adults and can dynamically adapt to the user’s actions and preferences. As benefits, PARO can reduce patient stress and indirectly of their caregivers, stimulate patients and contribute to the socialization part of their life. A recent study has shown that PARO can be very useful for improving quality of life of older adults with dementia and Alzheimer, affection and social interaction, reducing depression and anxiety—even for reducing pain medication usage [96]. Another comprehensive evaluation study highlights the potential usage of PARO as a pet therapy for older people with dementia while identifying barriers for its wide usage such as the fact that users’ needs and experiences are not properly taken into consideration and that it fails to meet the actual clinical needs [94]. Besides these, compared with nowadays social robots, PARO has no functionalities for requirements such as stimulating older adults’ memory or cognitive functions, increasing physical activities or aiding the caregiver with care information.

The Pepper robot developed by SoftBank is a humanoid robot featuring multi-modal communication able to recognize faces and basic human emotions, is capable of human interaction directly through conversation [97]. It can also exhibit body language, perceiving and moving around and is currently used in schools for child education or different businesses mainly as information point for users. Pepper is priced around €15,000 (acquisition price and subscription fee for three years) and has spawned multiple projects for developing new instances of its basic features such as ASIMO, COMAN or Enon and EU research projects such as CARESSES, CROWD-BOT or ANIMATAS. As for its underlying technology, Pepper uses a six-axis inertial measurement unit (IMU) sensor, microphones, cameras and 3-D sensor, laser sensing modules, loudspeakers, sonar sensors, infrared sensors, tactile sensors, bumper sensors and features an attached tablet for direct interaction and configuration. For the specific use–case of older adult care, Pepper was successfully used in healthcare and older adult-care facilities mainly as narrative-memory-based human–robot companionship [98] and medicine taking reminding, encouraging older adults to keep active and helping them keep in touch with family and friends [99]. Recent research approaches have used Pepper as older adults’ companion for suggesting personalized physical activities in the context of active aging [100]. The proposed solution uses deep learning methods on Pepper recorded information to classify the exercises and to schedule personalized physical activities. The robot is integrated in the context of a robot system named PHAROS which in addition to the robot component it contains a component for human exercises recognition which applies deep learning models on the data recorded by Pepper and a component which recommends physical exercises periodically considering the data from the agenda of the users. In [101] Pepper robot is used in the context of a system called Crowd of Oz (CoZ), an open-source system that allows conversational tasks. The objectives of CoZ are to enhance both the contextual and the social awareness of the workers, to manage the asynchronous nature of the workers during the conversational task and to support the task performance of the workers. The Pepper robot has also been used for the case of people that have special conditions such as schizophrenia or dementia during recreational or rehabilitation sessions [102]. Even though the humanoid robot can elicit simple instructions for simple activities such as physical exercises, numerous improvements are required in order to deploy the humanoid robots in the long-term care. Moreover, other aspects should be considered such as the motivation of the older adults or a sense of calmness from the clients’ side. Some benefits of the application of Pepper in hospitals [103] are the lowering of the stress levels and the contacting of the family members when the older adults are unable to contact anyone especially in critical situations when something happens to them. However, there are still further research directions that should be considered such as the recognition of a wider range of emotions.

The Nao robot is reported in several approaches as useful coaching assistant [104,105,106]. In [104] a particular use case for Nao is presented as an illustrative scenario for social robot driven intervention, an autonomous exercise tutor. The robot is capable to learn from a human, to generate feedback situations such as speed and amplitude adjustment, mirroring detection and no motion. However, the older adult participants did not prefer the intervention of the robots due to the poor social skills and because they perceived the sessions with the robots as one-to-one instead as a social event. In [105] a Nao socially-assistive humanoid robot is tested in the context of a smart home environment. Even though the results presented there suggest that the interaction between the robot and the older adults is not characterized by anxiety, the maintenance of high levels of enjoyment for the older adults for prolonged time periods is still a challenge. Nao was also used to detect the behavioral disturbances of the people with dementia [106]. The authors aimed to evolve the role of the humanoid social robot to a technological support tool that functions autonomously, to drive to the resident and alert the staff or distract the resident temporarily when a behavioral disturbance is detected.

There are several other social robots that are reported in the literature as potential solution for older adult care intervention. Zora is one of these and can be used in interventions for the care personnel and for the older adult-care institutions [107]. The results show various types of impacts, ranging from negative to positive. A part of the participants suffered from memory disorders and others required round-the-clock care. The impact on the care personnel differs very much from the impact on the older adults. In the case of the care personnel the impact was on dimensions such as the working atmosphere, the professional development and the competences, while in the case of the older adults the impact was on the physical activities, the older adults’ interaction and the sensory experiences. Authors of [108] consider a mini robot as support for a motivational decision-making system (DMS). The stimulating and the improvement of the interaction of the robot with the users is considered from various perspectives such as the performance of cognitive exercises and the performance of educational games. In the scenario described in that article the mini robot has two motivations, namely a social motivation and a relaxing motivation, and those two motivations must be in equilibrium while the robot interacts with a user. However, one drawback of that approach is that the robot is modeled using only two motivations and many other complex aspects should be considered when the users have specific health conditions. Another approach [109] proposes the learning of the social gestures through imitation through a humanoid robot called Tangy that is programmed to avoid the self-collisions and to generate arm trajectories that are collision-free. Even though the human demonstrators considered in that article do not have health conditions, the research considered in that article can be further adapted to stimulate the interactions of the older adults with the social robots. Another study that considers the application of social robots for older adults’ intervention is presented in [110] where the authors propose Stevie robot that is tested and validated on different categories of users such as the residents of a retirement community and the healthcare personal of that community. However, the capabilities of the robot should be extended in order to apply that robot in the case of the people with dementia or with different types of physical disabilities. A combination of more than one robot for developing behavioral intervention systems but focused on the children with autism spectrum disorders is proposed in [111]. The approach uses iRobiQ and CARO robots for providing training and support.

Table 3 summarizes the main approaches analyzed in the context of older adults’ robot driven coaching and intervention organized by the type of social robot intervention used.

### 2.4. Technology Limitation and User Acceptance

Older adults are keen to continue living in their own homes rather than move into residential institutions and the assistive technologies reviewed in the above sections have the potential of providing the need support in managing various problems of their daily life. However, there are specific challenges that need to be addressed for both technological development perspective and integration with the care models as well as regarding its acceptance by the older adults.

From the technological perspective, the nowadays older adults assistive services are shifting towards the use of objective monitoring using IoT sensors and sensors networks, but in general, they lack in personalization when it comes to addressing the older adults wishes and needs and are relying on dedicated healthcare resources for assuring the intervention and continuity of care. The assistive service models in the area of managing and carrying of older adults are focused on providing general information and awareness of the specific disease. Being overwhelmed due to daily life activities and carrying duties the end-users are no longer interested in general-purpose information, but they want personalized target support. In addition, most of today’s care process assessment is still relying on self-reporting of “perceived behaviors”, but this kind of models are proven not to be viable in the case of elders who mostly deals with problems such as forgetfulness and confusion and chaining mood and behavior. Recent IoT advancements and the development of miniaturized sensors have the potential of changing this situation, by enabling the remote and daily monitoring of important care aspects such as adherence to recommended therapy and lifestyle changes delivering more coordinated innervation through the means of social robots. In addition, this has the potential of improving the older adults’ engagement and adherence and timely communication among all parties involved in the care process. Moreover, there is a need of integrating within the nowadays care models novel technology solutions supporting the non-face-to-face interaction and follow-up of older adults, such as advanced robot-supported verbal communication tools with caregivers and patient and/or family/caretaker support for self-management, independent living and activities of daily living. All these limitations need to be systematically addressed to support the perspective shift for next-generation of coordinated care service models for older adults which are relying on ICT-based pervasive and objective monitoring of daily live functioning using advanced IoT sensors, quantifiable metrics of assessing the elders’ deviations signaling conditions decline and timely interventions supported by robots/tablets/avatars.

Acceptance of these novel technologies well as their daily usage may be challenging for older adults. As shown in the literature, several technology acceptances factors can be correlated with aging ranging from technology costs, privacy implications and usability up to social aspects such as the implications for family or friends [112]. Several acceptance models can be used to support the process as technology acceptance model (TAM), unified theory of acceptance and use of technology (UTAUT) or senior technology acceptance model (STAM) [113] by assessing indicators such as technology perceived usefulness, ease of use, experience or social implications. However, even these models cannot capture aspects such as user acceptance in time and miss key aspects in the older adult life such as cognitive decline and social isolation [114,115].

Monitoring older adults’ activities is the most common identified barrier for older adults’ acceptance since it involves breaking their privacy by installing smart devices such as wearables or sensor networks in their homes and collecting sensitive data [116]. Even that at beginning the older adults may not trust in these technologies, after using them they start to perceive them as important. A key role for accepting in home remote monitoring is attributed to family, friend and caregivers that can assist, train and support the older adult in embracing the change. Passive monitoring-based on IoT devices is the most suitable approach for smart home data collection while wearables technology seems to have a good acceptance ratio especially to well-educated older adults that appreciate the possibility of self-monitoring their health status [117]. In contrast, active monitoring such as monitoring older adults with cameras in their environment is raising major privacy and ethical concerns and consequently it encounters their reluctance of using such systems even though they are useful for users that have health problems such as dementia [118,119]. In domestic environments, sensor-based monitoring infrastructures usually require combining heterogenous sensors and devices for capturing information regarding the older adult behavior leading to another major issue for developing smart AAL systems, namely data heterogeneity. However, this issue was thoroughly researched leading to different solutions ranging from involving web semantics and sharing a common context model for building smart objects in heterogenous IoT networks [120,121] to defining and using ML and big data technologies to process time-series-based heterogeneous and distributed streams of data in a unitary approach [62,122].

The benefits of assistive technologies come from their core Artificial Intelligence (AI) components that use advanced ML techniques for taking decisions, alerting or providing support for doctors and caregivers. The user acceptance for these technologies relates to their understanding of the brought benefits and potential improvement for their quality of life. In general, older adults, doctors and caregivers agree and seem to be comfortable to us AI/ML technologies for assisting and improving the care process [123,124]. They are usually adapted to particularities of older adult’s health status such as mild dementia [83], medication and polypharmacy [88], cardiovascular problems [15] or physical activity [76]. However, the acceptance of such technology is usually influenced by factors such as age and education [125], while the adoption of intelligent technologies is corelated with the older adult loneliness and with the support provided by caregivers [114].

The use of social robots for older people care at home or in initialized care is a new technological trend for supporting the intervention in assistive care models. Existing studies for the social robots’ acceptance are relatively new and conclude that further research is required for assessing acceptance rate, but in the same time clearly identify the care areas where the robots can be successfully used as physical assistance, safety/monitoring and social companionship for an older adult [124,126]. Other surveys show that older adults have positive thoughts regarding the usefulness, utility, safety and trust of a social robot, while doctors and caregivers consider that the robot is a useful tool for rehabilitation [127]. In general, more positive acceptance is found after the users interact with the robot after a period, while the acceptance level is directly influenced by the robot’s social capabilities [128]. Other authors pinpoint that usefulness, adaptability, enjoyment, sociability, companionship and perceived behavioral control are important for the high acceptance rate of social robots [129]. One specific factor for accepting the social robot as a care companion is loneliness, the social robots may offer support and companionship especially at home [130].

## 3. Novel Integrated Robot-Based Care Services

In this section we discuss on the potential usage of the reviewed technologies for the development of advanced assistive and care services focusing on their features implemented and architectures.

### 3.1. Polypharmacy Management

Most older adults with comorbidities are taking several drugs per day being exposed to the negative effects of polypharmacy. Ensuring appropriate medication usage in this population is clinically important because of the significant risks for institutionalization and negative impact of drugs related problems on older adults’ wellbeing. Polypharmacy management includes the review of medication intake, identification of medication side effects and lack of adherence of patents thus it tends to be challenging in case older adults. For medical professionals it is difficult to properly assess the behavioral and psychological symptoms of the older adult patient and distinguish them from medication side effects, since changing is gradually and is likely to be multifactorial (also because the patients have difficulty describing the changing situation) while for the patient it is difficult to take the appropriate medication at the right time. A medication review is utmost important because unfortunately, due to age-related drug metabolism and other problems related to frailty (low vision and reduced psychomotor functioning among others) or due to transitions towards home healthcare, the older adult is at risk of experiencing drug-related problems. These problems are usually caused by drug use, drug choice and adverse reactions, interactions or contraindications. Drug related problems may occur in various steps of the medication process (from prescription till evaluation) and they are dependent on various actors in the chain of pharmaceutical care such as the physician, nurse, pharmacists or their accountable assistants and off course the patient himself.

**Figure 1:**
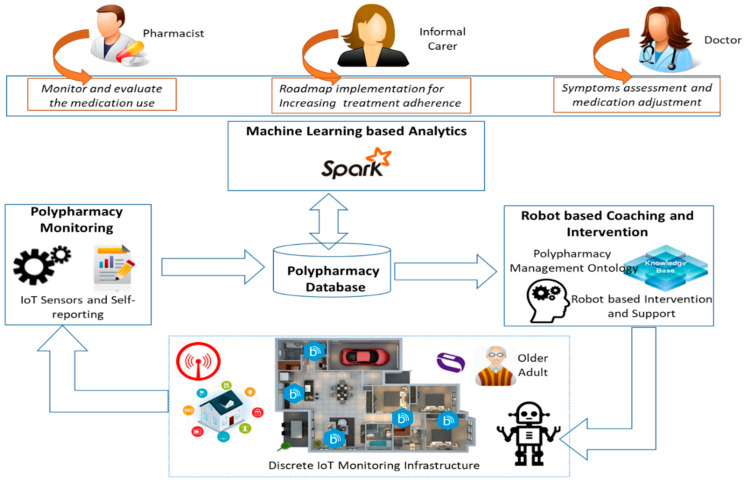
Assistive technologies use to implement the polypharmacy management service.

The polypharmacy management service (see Figure 1) aims improve the medication process for older adults by combining the objective monitoring by means of sensors with machine learning techniques to properly assess the medication use and potential side effects experienced and by leveraging on social robot/tablet to provide timely personalized support to older adult patients (i.e., medication reminders, direct link to caregivers or medical professionals, etc.). This service is related to the EU AAL MedGUIDE project [131], in which the authors are responsible to its technical implementation.

The polypharmacy monitoring deals with the acquisition of data from sensors that are deployed inside the older adult home targeting the monitoring of activity of daily life with a view of assessing the daily routine baseline and deviations and medication intake to asses adherence to the prescribed medication plan. The following types of activities of daily living were identified as relevant and on-the-shelf commercial sensors, including devices associated with the environment in which the older adult patient lives were used for their monitoring:

- Community mobility—refers to outdoor activities;
- Feeding—refers to the activities of preparing and eating food;
- Functional mobility—refers to indoor activities;
- Total hygiene—refers to the toilet visits and showering activities;
- Sleeping—refers to overnight sleeping and afternoon naps

Motion sensors and iBeacon tags are placed around the home such as on the refrigerator door or on the bed, to monitor and deduce older adults’ activities (see Table 4).

For medication intake monitoring on-the-shelf pillboxes is used. In this case, the pillbox will not be used as a simpler organizer or a locked box with a daily alarm but will enable the assessment of adherence level to the medication plan and the report that information back to a doctor, pharmacist or caregiver.

Machine learning-based analytics leverages on big data techniques to process the heterogeneous and distributed streams of monitored data to establish the baseline daily life activities of older adult and to detect in real time events that represent changes, either sudden or gradual, in patients’ activity routines which may signal progression of his symptoms, wellbeing decline or side effects of medication [132]. The recent advancements in sensing technologies, IoT and the prevalence of miniaturized, affordable sensors and smart objects, will led to an “explosion” in contextual big data that may be used for improving the older adults with dementia care and treatment. To efficiently exploit the large amount of historical monitored data machine learning algorithms may be used to extract new knowledge and correlation between unrelated daily life activities events which may represent deviations from original older adult patient baseline.

**Figure 2:**
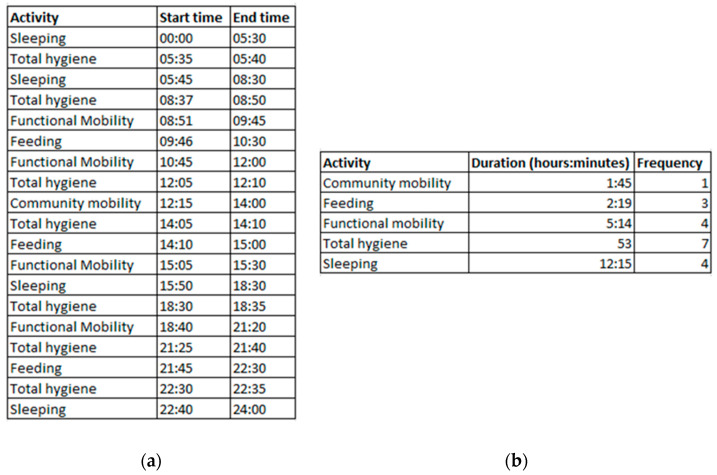
(**a**) Example of monitored older adult activities in a day; (**b**) features extracted and used in machine learning.

The data sensor records collected throughout a day are further aggregated and the relevant features are extracted to identify the daily sequence of activities for each older adult (see Figure 2).

**Figure 3:**
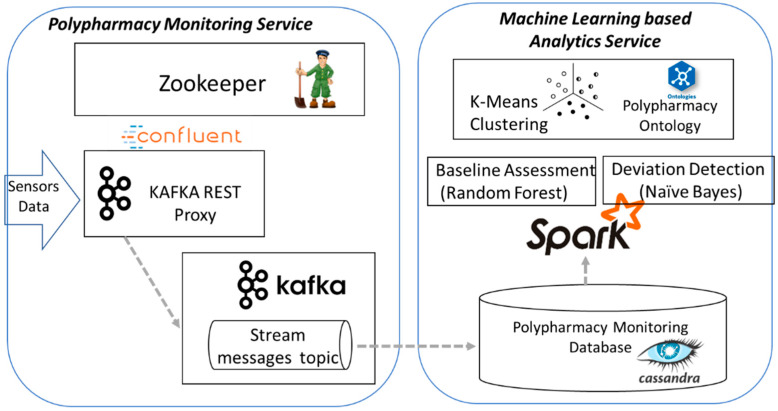
Sensor data management and machine learning (ML)-based analytics.

The sensors data flow management is achieved using the following technologies (see Figure 3): (i) Zookeeper [133] as centralized service used for maintaining configuration information, distributed consistent states and synchronization, (ii) Kafka [134] for building real-time data streaming pipelines to be integrated in the master data set and (iii) Cassandra [135] database for storing time series data from sensors.

Baseline assessment techniques aim to identify the routine of an older adult for the entire day, i.e., the daily activities that s/he will normally carry out. Features are extracted from the daily monitored data, and for each type of activity considered, an input for the random forest classifier is provided, which works by building a set of decision trees. Each decision tree is trained on a subset of the training data set. During the testing phase, each decision tree part of the random forest votes for the class to which a test instance belongs (i.e., baseline or not); the class with the most votes is assigned to the associated test instance [136].

Deviation detection techniques seek to detect changes in older adult daily routines that may represent potential side effects of medications s/he had taken, as corelated with the information acquired using the Table 4 sensors infrastructure and pillbox monitoring. A significant deviation from the baseline may be classified, for example, if the total time and frequency corresponding to at least one activity type performed by the older adult on a specific day is higher or lower than a pre-defined threshold as compared to the same activity type registered in the baseline. For this technique, naïve Bayes classifiers can be applied to compute the probability of performing a specific type of activity at a given moment, considering the baseline behavior as a set of conditional probability models. 

To correlate detected deviations from potential side-effects of drug–drug interactions, a drug–drug interactions domain ontology is used [137]. This ontology models the pharmacological effects of drugs; the pharmacodynamics actions of drugs; the mechanisms by which these actions are performed; the processes of absorption; transportation, distribution, metabolism and elimination of drugs; the recommended dose and the interactions between drugs. Concepts of the ontology are used to label and annotate the significant deviations detected with potential side effects of drug to drug interaction taken form ontology. Then, a clustering algorithm such K-means is trained and then used to cluster similar days containing significant deviations from the baseline and the results of the clustering algorithm are used to correlate future monitored days with potential drug–drug interactions. Each cluster will contain similar annotated days and the label of the cluster is given by the annotation (i.e., drug–drug interaction and its adverse effects) of the cluster’s centroid.

Robot-based coaching and intervention provides personalized care and coordinated guidance, motivation and support for the older adult patient and associated informal caregivers aiming to increase their adherence to the medication plan. An interactive social robot may be used that can speak, respond and receive and send messages to any kind of electronic device (candidate robots: James robot [138], KOMP/AV1 robots [139], etc.). The output of the machine learning analytics will be used for personalizing the robot-based interventions. To achieve this, we pre-define and use robot activities configuration templates and scripts that are associated with each type of identified definition. They will be defined using drag and drop symbol-based programming language and will be integrated with the robot to automatically boot strap and setup the coaching processes fitting older adults’ needs, wishes and post discharge recommendations. This will positively impact their adherence to medication plan and will overcome problems which may lead to re-hospitalization. At the same time, the robot can be used to deliver roadmaps and list of actions for better polypharmacy management by implementing the doctors and pharmacist recommendations. Predefined step-by-step video or audio instructions in conducting various lifestyle changes activities or reminders for taking medications delivered using the robot. This may include care feedback and anticipatory guidance for transitional care allowing the patient or the caregivers to repeat information back to confirm understanding (“teach-back”). In addition, personalized motivation slogans and sayings will be pre-registered and used to achieve a high degree of personalization of the care of older adults as the symptoms progress helping them to follow the doctors’ recommendations. Social robot usage will increase the independence of the older adults allowing them to connect on-demand with the doctor or informal caregiver via scheduled virtual video meetings.

**Figure 4:**
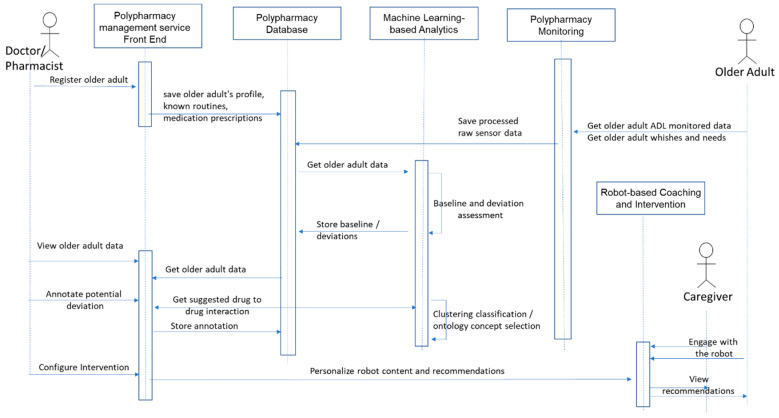
End-user interactions and data flows among modules of the polypharmacy management service.

Figure 4 shows the main data flow among components as well as the interaction of end-users with the polypharmacy management service as well as the interactions between the service modules. The doctor/pharmacist may register the older adult with the service and introduce relevant information known routines, active drug prescriptions—including the drugs that the older adult takes, the dosage, the intake moments and the duration of the treatment. They may check the older adult’s medication adherence for a selected date, the daily baseline and potential deviations as well as the machine learning-based suggestions for potential deviations. In addition, they play an important role in robot-based intervention configuration by annotating the deviations with potential side effects of the drug–drug interactions and providing personalized recommendations and content to be played by the robot. The older adult activities of the daily living are continuously monitored, may provide his/her wishes and needs concerning the service and receive the recommendations, instructions to follow via engaging with the social robot companion. The caregiver may supervise the interaction of an older adult with the robot and at the same time may view the recommendations to be implemented.

### 3.2. Social and Cognitive Activity Engagement

Social assistive robot-based systems featuring sensor-based monitoring, activity and cognitive games and social networking capabilities can be used to stimulate the physical, cognitive and social conditions of people by consolidating their current condition and/or restraining deterioration of their cognitive state [140]. To improve their quality of life while independent living at home, a social robot can provoke the older adult in playing games and perform social activities considering their current state, wishes, needs and preferences.

The older adults’ monitoring is based on a combination of seniors and virtual sensing devices (self-reporting condition or memories) used to determine the older adults’ activity levels, sleeping patterns/quality and mood as well as their metal condition and relevant information to achieve a more personalized interaction with the robot. The robot device can be used as an intelligent hub for collecting and pushing data into the cloud for further analysis and assessment. The monitored data will be stored in a data storage enacting its future processing to allow contextualization of the robot-based activities.

The assessment will be centered on detecting older adults either sudden or long-term decline in physical, social or cognitive activities. Machine leaning techniques can be employed to mine the monitored data, for translate the older adult’s past and present state into estimates of the future thus, obtaining a reference to the potential decline well-being (i.e., long-time trends, seasonal and irregular components, etc.). Motivation to conduct specific activity is challenging and will be implemented using gamification, providing goal and reward scores and social networking applications. Physical and mental stimulation and prevention of cognitive decline will be provided through interactive gamification.

A certain degree of personalization can make cognitive and physical activities more social and enjoyable for the older people by an innovative combination of visual games and applications and a robot companion. The robot modules will not be merely standalone applications, but will be contextualized, filtered and integrated to give personalized experience of a smart and seamless environment. The personalized filtering will facilitate social participation of the older person, reminding scheduled upcoming activities and visits, either through direct communication or through displaying them on tablet and/or flat screen TV. The robot will encourage them and follow-up on a wide range of activities, which can be performed individually or in groups and both indoors and outdoors. Publicly available exercise games and content will be used/adapted to interface with the robot and will not require log in from older adults being offered in a full-kiosk mode to avoid accidentally closing or exiting the application and improving the app has visible and clear appearance.

**Figure 5:**
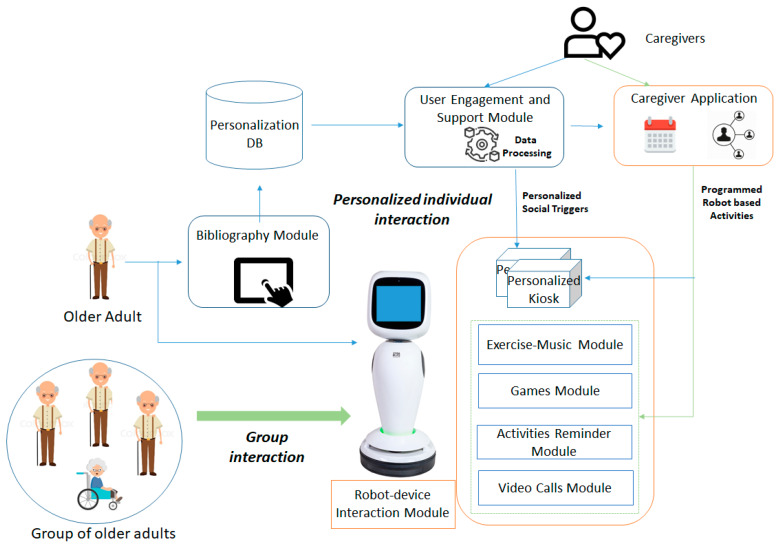
Personalization of the robot-based interaction.

The conceptual architecture of this system, to be developed in the context of EU AAL ReMind project [141], is presented in Figure 5 and defines the two type of interaction paths between the older adult and the robot (Zora James robot): (i) individual personalized interaction—one-on-one interaction of an older adult with the robot and (ii) non-personalized interaction—more related to the group of older adults interaction with the robot (i.e., fitting the care facility scenarios).

The personalized interaction path is supported through the bibliography module, which is responsible to acquire and store various biography information (i.e., memories) to be used then for defining the older adult customized interaction. The biography data are stored in a personalization database, on top of which the user-engagement and support module will run analytics to detect the right social triggers for personalizing the older adult—robot interaction. The personalization will be carried out both with regards to the type of robot-based applications to be triggered at a specific moment of time and with the content the applications will leverage. As result personalized kiosk of robot-based applications will be created customized to the biography information of the older adult. The caregiver (CG) will be able to filter the inferred social triggers and using the caregiver application will set up appointments for robot driven activities using a calendar-based application. At the same time, it will be able to put in contact or connect the older adult with its social network (i.e., family members, friends, etc.) to carry out different social activities.

On the robot various modules are implemented and used in correlation with the activities the older adult may conduct to increase its activities levels and timely address the potential cognitive decline. The exercise–music module will be able to play entertainment content such as music, video or TV or it will provoke the older adult to conduct physical activities such as follow the robot. In addition, it will be able to play videos of older adults performing physical activities tailored to the older adult’s physical condition and will engage them in such in physical activities by providing instructions and encouragement. The games module will be featuring games that can be played with the help of the robot. Games such as bingo, proverb quiz, packing my suitcase, etc. will be considered with the robot being able to play the role of the game host or caller. The activities reminder module will be able to remind to drink water and take medication as planed and will provide reality orientation activities by providing information on weather, news, upcoming events and daily meals. The video calls module will be able to set up video calls for the older adult with members from its social network based on previously made appointments in calendar. The interaction between the older adult and the robot is facilitated by the robot–device interaction module, featuring vocal commands and tactile-based interaction.

**Figure 6:**
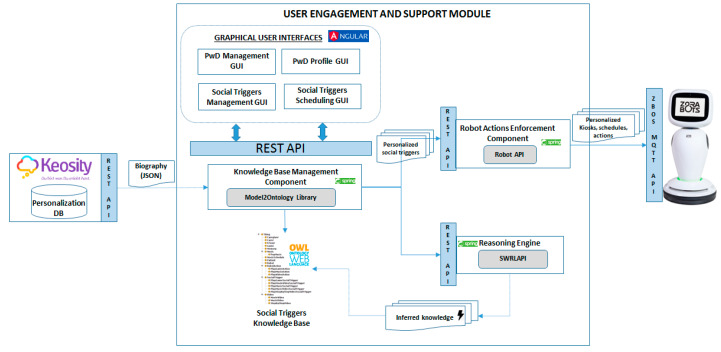
User-engagement and support module implementation.

The user-engagement and support module implements and runs analytics on top of the data collected by the biography module to understand the social triggers for older adult’s engagement with the robot and to create personalized kiosks according to the older adult profile and biography, to be enforced on the robot. The personalization will be achieved both in regards with the type of robot-based applications to be triggered at a specific moment of time and with the content that the applications will use. Figure 6 presents the internal architecture and technologies used for implementing this module.

The following internal components will be implemented. Knowledge Base Management component is responsible for performing create/read/update/delete (CRUD) operations on the social triggers’ knowledge base using the Model2OntologyLibrary [142]. It is also responsible for interacting with the Keosity application [143] through its dedicated representational state transfer (REST) application programming interface (API) to extract the biography of the older adult. The Model2Ontology Library it is a library defined by the authors is providing a very light interface for accessing the knowledge in the form of ontology and reduces the code complexity. It offers one-line methods for performing basic operations (create, update, delete, find). It uses reflection to parse the Java entities, hiding in this way the code complexity needed by APIs like Jena [144] and Ontology Web Language (OWL) API [145] to perform operations on ontologies, at the same time, benefitting from the performance and scalability properties offered by these. Furthermore, it is the first library that offers the functionality of generating semantic ontological model from an object-oriented model.

**Figure 7:**
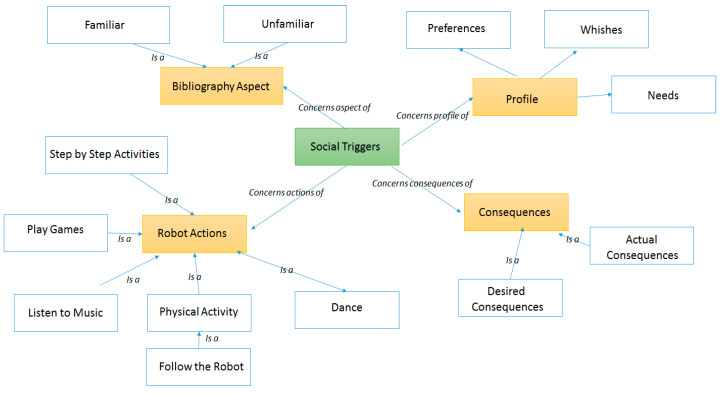
Proposed older adult–robot engagement and motivation ontology.

The social triggers knowledge base is the data model of this module being implemented as an OWL ontology which contains the concepts and rules-based on which the older adult specific data are analyzed. The social triggers knowledge base will model four main facets of the older adults as core sub-ontologies (see Figure 7 for the ontology design model):

- Bibliography aspects—which may be familiar or unfamiliar and it is collected using the bibliography module;
- Personal profile aspects—which concern his/her preferences wishes and needs being also provided by the bibliography module;
- Robot-based Actions—potential actions in which the older adult may be engaged with the robot;
- Consequences—the actual and desired result of conducting a specific activity with the robot;

The reasoning engine is based on an inference engine allowing to run reasoning rules on the ontology considering the older adult specific data to infer personalized triggers that will be further enforced on the robot. It is based on tools like Pellet [146], Jena and D2RQ [147] and will run and evaluate the defined rules for assessing older adult specific triggers by using the data feed by the bibliography module. Two types of rules could be defined and used on the user-engagement and support ontology classes and associated individuals: reasoning rules and query rules. The reasoning rules are written in the semantic web rule language (SWRL) [148] language and used to infer new social triggers information or knowledge out of the ontology. The SWRL rules are injected into the ontology and used to reason about ontology individuals in terms of specific concepts, object and datatype properties. Rules are written in the form of an implication between an antecedent (body) and consequent (head). Both the antecedent and consequent consist of multiple atoms conjunctions. The SWRL rules are evaluated by a reasoning engine in our case the Pellet reasoner. The query rules are written in semantic query-enhanced web rule language (SQWRL) [149]. They are language is data-oriented in the sense that it only queries the individuals held in the ontological models and makes no inferences. In the case the reasoner is started, the queries can return not only data that exists physically in the ontology, but also data that is inferred.

For example, the SQWRL query from Table 5 selects the music content available on the robot to which the older adult is familiar with; in this case, a music file being loaded on the robot if the name of the singer associated to the music file is retrieved in the memories provided by the older adult using the biography module.

In the same table an example of SWRL rule is provided that infers whether an older adult has knowledge (i.e., is familiar) with some music content available on the robot by determining if the name of the singer associated to the music file is retrieved in the stored memories. By executing the rule, a correlation between the older adult and the specific singer is established using the has Knowledge Of Music object property.

The robot actions enforcement component converts the older adult personalized triggers into specific robot actions and personalized kiosks that are further run on the robot. The communication with the robot is performed through a message queuing telemetry transport (MQTT) API [150]. To create a new kiosk, the inferred information about the personalized social triggers is used as a filter to search for specific content in the robot’s files system by means of an executioner filter. The filtering result is sent to a kiosk builder that creates a new kiosk object which is sent using MQTT connection and uploaded to the robot.

## 4. Conclusions

This study provides a comprehensive survey of smart environments and robot assistive technologies by identifying the main research problems and technologies limitation and highlighting the current status of their development. The goal was to analyze the foundation for implementing age-friendly care services and for supporting the independent living of older adults at home. The study is organized in three directions: monitoring daily activities, machine learning for behavior assessment and social robots-based intervention. The survey shows that even though various technologies and techniques do exist in the AAL domain, future research is required for matching these onto the specific needs of older adults and their living context and increase their level of adoption. Finally, we discuss on potential usage of these technologies, in the context of two innovative care services, namely polypharmacy management and social and cognitive activity engagement. These care services are based on the findings and developments made in ongoing H2020 Ambient Assistive Living projects and showcase the potential of smart spaces, machine learning and social robot-based systems for improving the quality of life and care processes of older adults. As future steps, we plan to integrate the proposed big data polypharmacy management infrastructure into the H2HCare AAL project platform for developing an older adult post-discharge monitoring and follow-up service that will assess the adherence to the prescribed post discharge lifestyle changes recommendations and medication plan of an older adult and will offer support to him/her through the KOMP/AV1 social robots. In the same fashion, we plan to further develop and integrate the social and cognitive activity engagement service as part of the ReMember-Me and ReMIND AAL projects for developing solutions for stimulating the physical, cognitive and social conditions of older adults through the James robot.

## Figures and Tables

**Table 1:** Ambient assistive living (AAL) physical sensors and data provided.

Available AAL Sensors	Type of Monitored Data	Approaches
**Wearable sensors**		
Body temperature sensors, biosensors for monitoring vital signs	Body temperature, physiological attributes (e.g., heart rate, temperature, blood pressure, respiration rate, etc.)	[15,16,17,18,19,24,29]
Motion sensors such as accelerometers, gyroscope, magnetometers, passive infrared sensors, GPS, GSM, active badge systems	Movement, indoor/outdoor location, position, posture and gait	[15,16,21,22,25,26,27,30,32,33,34,35,36,37]
Photosensors, color sensors, acoustic sensors (i.e., microphones), etc.	Light levels, sound and audio	[22,24,25,26,30]
Body sleep sensors	Sleep levels, patterns, intensity, etc.	[20,22,23,30,31]
**Non-Wearable sensors**		
Touch sensors	Touch (allow interaction with smartphones and tablets or home appliances)	[54,55,56,57,58,59,60]
Force/floor sensors	Falls and movement (walking, standing, sitting, etc.)	[14,45,47,48,49,50]
Pressure pad sensors	Surface pressure measurement (e.g., bed pressure mats)	[39,40,41,42,53]
Video sensors (e.g., various cameras)	Visual context (e.g., keep track of daily living activities performed by the older adult, locating the older adults in house)	[43,44,45,46]
Acoustic sensors	Fall-detection	[48,49,50]
Ambient sensors (temperature, appliances, toilet)	Ambient temperature, usage time duration of an equipment, Toilet-usage frequency	[14,38,51,52]
Contact sensors, magnetic switch	Open/close the office desk, open/close the TV, open doors, windows, etc.	[14,38,52]

**Table 2:** Potential usage of sensors.

Type of Assessment		Sensors	Potential Usage
Physiological	Stress/anxiety level	Wearable sensors for pulse rate, temperature, blood pressure	Stress or anxiety detection -> Play music as intervention
Daily life activities assessment	Sleeping	Bed Pressure Sensors	Sleeping problems detection
	General Activity level	Motion Sensors	Lack of physical activity -> individual training intervention
	Food intake	Devices embedded sensors	Intake problem -> intervention by reminding to eat, drink water, etc.
	Medication Intake	IoT Pillbox	Medication plan adherence problem -> intervention by reminding to take medication according to the prescription plan
Social Interaction	Physical interaction	Camera and image processing and Voice recognition	Video-based communication to support mediated connection
	Virtual interaction	Social network-based monitoring	
Cognitive	Automatic Reminders	Voice recognition	Memory stimulation using biography
		Personalized Information	News/weather feed
Safety	Safety Assistance	Fall detection sensors	Send of alerts/notifications

**Table 3:** Social robots’ intervention approaches in research literature.

Social Robot	Approach	Conditions	older Adult Interventions
Nao	[104,105,106]	cognitively healthy older adults; persons with dementia/Alzheimer’s	detection of behavioral disturbances; physical exercises tutoring, recreational activities; physical training
Pepper	[99,100,101,102,103]	cognitively healthy older adults; crowd workers; people with schizophrenia or dementia	detection and classification of physical exercises; stress management, companion for older adults; rehabilitation recreational activities; sentiment analysis; narrative-memory-based human–robot companion; medicine taking reminding, encouraging older adults to keep active and social stimulation
PARO	[94,95,96]	older adults with dementia	pet therapy; reduce patient stress; social interaction, reducing depression and anxiety
Stevie	[110]	care house residents and caregivers	care support, entertainment, cognitive engagement, social connectivity
iRobiQ & CARO	[111]	children that have autism disorders	social training, emotions analysis
Zora	[107]	older adults with memory disorders	stimulating older adults through exercises and interaction
mini & Tangy	[108,109]	cognitively healthy older adults	educational games; imitation learning

**Table 4:** Types of sensors installed in the home and the monitored daily life activity.

Sensor Names	Installation Place	Monitoring of	Daily life Activity
Bed sensor	Bedroom	Sleeping pattern of an older adult in terms of period and continuity	Sleeping
Fridge sensor	Kitchen	The number of times the fridge has been opened by the older adult	Feeding
Motion sensor	Kitchen	The older adult’s activity in the kitchen	Feeding
Entrance sensor	Entrance	The number of times the entrance door has been opened or closed	Community mobility
Motion sensor	Entrance	Whether the older adult has left or entered the home	Community mobility
Motion sensor	Living room	How much physical activity is performed in the house	Functional mobility
Motion sensor	Bathroom	The number of times the older adult has been to the toilet	Hygiene

**Table 5:** Example of social triggers assessment rules.

Type	Social Trigger Assessment Rule
SWRL rule	Patient(?p) ^ hasId(?p, ?id) ^ swrlb:matches(?id, 1) ^ hasMemory(?p, ?m) ^ hasDescription(?m, ?d) ^ hasRobot(?p, ?robot) ^ hasPlayMusicAction(?robot, ?action) ^ hasMusic(?action, ?music) ^ hasSinger(?music, ?singer) ^ hasId(?music, ?musicid) ^ swrlb:contains(?d, ?singer) -> sqwrl:select(?musicid)
SQWRL query	Patient(?p) ^ hasId(?p, ?id) ^ swrlb:matches(?id, 1) ^ hasMemory(?p, ?m) ^ hasDescription(?m, ?d) ^ swrlb:contains(?d, \”Michael Jackson\”) ^ hasRobot(?p, ?robot) ^ hasMusic(?robot, ?music) ^ hasSinger(?music, ?singer) ^ swrlb:contains(?singer, \”Michael Jackson\”) -> hasKnowledgeOfMusic(?p,?music)
